# Use of ecstasy and other psychoactive substances among school-attending adolescents in Taiwan: national surveys 2004–2006

**DOI:** 10.1186/1471-2458-9-27

**Published:** 2009-01-21

**Authors:** Wei J Chen, Tsung-Chieh Fu, Te-Tien Ting, Wei-Lun Huang, Guang-Mang Tang, Chuhsing Kate Hsiao, Chuan-Yu Chen

**Affiliations:** 1 Institute of Epidemiology, College of Public Health, National Taiwan University, 17 Xu-Zhou Road, Taipei 100, Taiwan, Republic of China; 2 Department of Public Health, College of Public Health, National Taiwan University, 17 Xu-Zhou Road, Taipei 100, Taiwan, Republic of China; 3 Department of Psychiatry, College of Medicine and National Taiwan University Hospital, National Taiwan University, 7 Chung-Shan South Road, Taipei 100, Taiwan, Republic of China; 4 Division of Mental Health and Substance Abuse Research, National Health Research Institutes, 35 Keyan Road, Zhunan, Miaoli County 350, Taiwan, Republic of China

## Abstract

**Background:**

With the backdrop of a global ecstasy epidemic, this study sought to examine the trend, correlates, and onset sequence of ecstasy use among adolescents in Taiwan, where a well-established gateway drug such as marijuana is much less popular.

**Methods:**

A multistage probability survey of school-attending adolescents in grades 7, 9, 10, and 12, aged 11–19 years, was conducted in 2004, 2005, and 2006. A self-administered anonymous questionnaire elicited response rates ranging from 94.3% to 96.6%. The sample sizes were 18232 respondents in 2004, 17986 in 2005, and 17864 in 2006.

**Results:**

In terms of lifetime prevalence and incidence, ecstasy and ketamine by and large appeared as the first and second commonly used illegal drugs, respectively, among middle (grades 7 and 9) and high school students (grades 10 and 12) during the 3-year survey period; however, this order was reversed in the middle school-aged students starting in 2006. Having sexual experience, tobacco use, and betel nut use were factors consistently associated with the onset of ecstasy use across years. The majority of ecstasy users had been involved in polydrug use, such as the use of ketamine (41.4%–53.5%), marijuana (12.7%–18.7%), and methamphetamine (4.2%–9.5%).

**Conclusion:**

From 2004 to 2006, a decline was noted in the prevalence and incidence rate of ecstasy, a leading illegal drug used by school-attending adolescents in Taiwan since the early 2000s. The emerging ketamine use trend may warrant more attention in the future.

## Background

Ecstasy, the street name for the hallucinogenic stimulant 3,4-methylenedioxy-methamphetamine (MDMA) [1], is a drug typically associated with dance clubs and rave movements. Since its introduction in Europe in the 1980s [2], ecstasy has gradually gained in popularity across many parts of the world, not only in industrialized nations [3-9] but also in less developed countries such as Taiwan [10], Hong-Kong [11], Turkey [12], and South Africa [13]. One noteworthy fact is that ecstasy has broken the boundary of typical drug scenes, so access to ecstasy (or other illegal drugs) is remarkably increasing, particularly among less experienced drug-users [14]. Growing concerns have been raised about ecstasy use during adolescence given that brain development during this period is more vulnerable to drug-related deficits [15].

Cumulative evidence, primarily derived from purposive sampling, e.g., known ecstasy users [16-20] or high-risk groups [6,10,21-23], indicates that polydrug experience is very common among ecstasy users [24]; however, whether such an observation can be generalized to ecstasy users in general remains unclear. Although some researchers have attempted to probe ecstasy problems using representative samples, such as school-attending adolescents in Norway [2] and Turkey [12], community youth in Germany [25], compulsory military conscripts in Spain [26], and nationally representative samples of youths [27] or household surveys [14,28] in the U.S. and Australia [29], few representative studies have been conducted in Asian countries.

In Taiwan methamphetamine was considered as the most common illegal drug used by adolescents prior to 2000, with a lifetime prevalence ranging from 0.65% in a national survey of students aged 13–18 years in 1996 [30] to 4.9% in vocational high school attendants aged 16–18 years [31]. Marijuana, the most consumed and well-established gateway drug in the western society [32], was rarely available in Taiwan until 1995 [33] and the prevalence was reported to be as low as 0.32% to 0.59% in school-attending adolescents [34]. Since the first official seizure in 1998 [33] the prevalence of ecstasy has been steadily rising; some small scale surveys have indicated that methamphetamine seems to have been replaced by ecstasy as the leading illegal drug in adolescents [34,35]. Given that the pattern and trend of drug problems may vary greatly with macroenvironment (e.g., culture), a better understanding of ecstasy epidemiology in different societies is greatly needed to provide effective prevention or intervention. Nevertheless, to date, it remains unclear which factors are associated with the onset of ecstasy use and what the role of ecstasy is in the drug transition of adolescents, particularly in regions where a well established gateway drug such as marijuana is much less popular.

Based on three consecutive years of a national school survey in Taiwan, the present study aimed to examine (i) the trend of cumulative and onset use of ecstasy among school-attending adolescents; (ii) possible changes in the sociodemographic profiles and deviant behavioral correlates with initial use of ecstasy over time; and (iii) the secular variation in initiation sequences of ecstasy in relation to other psychoactive substances over time.

## Methods

### Study sample

This study was based on the National Survey of Illegal Drug Use among Adolescents (NSIDA), a cross-sectional survey of behavioral and psychological problems, drug experiences, and life events of school-attending adolescents of grades 7, 9, 10, and 12, aged 11 to 19 years, in Taiwan. During each academic year in 2004–2006, the NSIDA team drew nationally representative samples from schools via multi-stage, random cluster sampling procedures. The sampling of the participants has been described in detail elsewhere [36,37]. Briefly, a sampling strategy was developed to cover three types of schools, i.e., middle school (grades 7 and 9), regular high school (grades 10 and 12), and vocational high school (grades 10 and12), in seven major geographic clusters in Taiwan [38]. The seven groups were derived through cluster analysis on five urbanicity-related variables (including population density, population ratio of people with college or above educational levels, population ratio of elder people over 65 years old, population ratio of people of agriculture workers, and the number of physicians per 100,000 people), with cluster 1 representing the highest and cluster 7 the least urbanicity.

On the basis of aggregate lists obtained from the Ministry of Education, a total of 133 schools were randomly selected each year, with 49 from middle schools (selection probability = 7%), 42 from high schools (13%), and 42 from vocational schools (27%). When school directors were unwilling to allow their school's participation (one vocational high school in 2004, three vocational high schools in 2005, and three middle schools, one regular high school, and two vocational high schools in 2006), a replacement school of the same type was selected from the same region. Within each school, two classes were randomly selected from the first and the third grades. All the students in sampled classes were eligible to participate in the survey. After written permission was obtained from the students, they were asked to complete a questionnaire. Since the questionnaire was anonymous, parental permission was not obtained. The participation rates for the three survey years were 94.3%–96.6% for middle school students, 91.5–93.4% for regular high school students, and 88.3%–94.0% for vocational high school students. The numbers of school-attending respondents for the NSIDA were as follows: 18562 in 2004 (7131 for middle schools, 6049 for regular high schools, and 5382 for vocational high schools), 18334 in 2005 (6934 for middle schools, 6169 for regular high schools, and 5231 for vocational high schools), and 18146 in 2006 (6317 for middle schools, 6022 for regular high schools, and 5807 for vocational high schools).

Because the focus was adolescent drug experiences, the present study excluded (i) respondents whose responses were missing drug and self-information items: 299 in 2004, 220 in 2005, and 100 in 2006; these students were not different from those included in the distribution of age, sex, or grade; (ii) respondents who reported having used a fake drug (12 in 2004, 5 in 2005, and 4 in 2006); and (iii) respondents whose age were < 11 or ≥ 20 years (22 in 2004, 128 in 2005, and 178 in 2006). The final sample for this study included the remaining 18232 respondents in 2004 (7083 middle school, 5941 high school, and 5208 vocational school students), 17986 respondents in 2005 (6836 middle school, 6071 high school, and 5079 vocational school students), and 17864 respondents in 2006 (6172 middle school, 5961 high school, and 5731 vocational school students).

To take the differential sampling probability and non-response rate into account, a sampling weight was derived for each participant. The comparisons between the weighted samples and the corresponding school populations showed no statistical differences in grade, gender, and school types; therefore, our weighted estimates may be extrapolated to the entire population of school-attending adolescents in Taiwan during the study period. Over the three-year assessment, the weighted percentage of males (51.98%–52.26%) was slightly greater than that of females (48.02%–47.74%), which is an accurate reflection of the composition of more males than females in the student population of vocational high schools. For ease of presentation, the seven major geographic clusters were collapsed into four major regions (regions 1 and 2 as North, 3 and 4 as Central, 5 and 6 as South, and 7 as East part of Taiwan) in this paper.

### Data collection

Data collection was primarily conducted during regular class time by 1–2 research assistants. Regular and vocational high schools were approached during the spring semester and middle schools during the autumn semester each year due to logistical considerations. During the 2004 spring semester, the survey assessments were executed using self-administered, paper-and pencil, anonymous questionnaires requiring a mean duration of about 20 to 30 minutes. In the 2004 fall semester, a web-based anonymous questionnaire, as described elsewhere [34], was available to facilitate the data collection nationwide. After 2005, the web-based questionnaire was implemented universally, and subsequently only when a school did not have sufficient computer room was the paper-and-pencil questionnaire applied, with the proportion being 8.0% in 2005 and 6.8% in 2006. Each participant received stationery incentives valued no more than 20 New Taiwan Dollars from the NSIDA team for a completed assessment (1 USD ≅ 30 NTD). The study was reviewed and approved by the Committee of Scientific Research and Human Rights Protection of College of Public Health, National Taiwan University.

### Measures

A fixed sequence of standardized pre-worded and pre-coded survey questions was designed to obtain information regarding each potential participant's sociodemographic background, problem behaviors, substance use experiences, and life events. The measures on substance use covered lifetime experiences of tobacco, alcohol, betel nut, and nine kinds of illegal drugs or inhalants (including ecstasy, ketamine, marijuana, angel dust, gamma hydroxybutyrate [GHB], methamphetamine, flunitrazepam [so-called FM2], heroin, and glue). Although selling tobacco or alcohol beverages to individuals under 18 years is prohibited by law in Taiwan, the enforcement of this regulation varies among shops. Betel nut (or areca nut) is a mild central nervous system stimulant widely used in Asia. Its active principle is the alkaloid arecoline, which stimulates both the parasympathetic and sympathetic nervous systems with dose-dependent responses [39]. For each substance endorsed, respondents were asked further drug-specific questions regarding age of first use, situation of first use, average frequency of consumption, and recency of use. Alcohol use was defined in our questionnaire as consuming a cup of alcoholic drink (e.g., about 100 c.c. beer) at least once. In the present study, the initial ("incident") use was defined as drug initiation within 12 months of assessment (i.e., age at survey – age at first use of any particular drug < 1).

In addition, a "bogus" drug was added to test the validity of self-reported substance use. In a two-week test-retest reliability study of the questionnaire used for this study with 67 middle school students, the drug-specific *Kappa *for readily available substances were excellent (0.81 for alcohol drinking, 0.93 for tobacco smoking, and 1.0 for betel nut chewing) and the percent of agreement was close to 100% for the items on illegal drugs [34].

### Data Analysis

Due to the multistage probability sampling procedures employed in the NSIDA, survey analysis procedures that allow for differential weights were used to estimate the use rate of a variety of substances. The associations linking sociodemographic characteristics and behavioral correlates with ecstasy use were examined by means of logistic regression analysis. The odds ratios (ORs) and 95% confidence intervals were derived taking into account sampling weights (e.g., to compensate for variation in sample selection probability) and complex sampling design via the Taylor series method [40]. The Cochran-Armitage trend test was used to examine the secular trend of substance use prevalence over years, while the Jonckheere-Terpstra test for trend was used to examine the ordered differences between the age at onset of other substance use and that of ecstasy use (younger, the same age, and older) over the years. All the statistical analyses were conducted using the Proc SURVEYFREQ and SURVEYLOGISTIC of software package SAS, version 9.1 (SAS Institute Inc., Cary, NC).

## Results

For middle school students (grades 7 and 9), a decreasing trend in lifetime use prevalence over the years was found in tobacco, betel nut, ecstasy, and GHB (Additional file 1: Table 1, upper panel). Among high school students (pooling regular high with vocational high school students together; grades 10 and 12), a similar decreasing trend was shown in psychoactive substances such as tobacco, betel nut, ecstasy, ketamine, marijuana, flunitrazepam, and GHB (Additional file 1: Table 1, lower panel). In general, participants' reported ages at first use were younger for readily available substances than for illegal drugs or inhalants, younger for middle school students than for high school ones, and increased across the years for all kinds of substances. Ecstasy and ketamine, by and large, remained the most and second most commonly used illegal drugs, respectively, during the 3-year period for both middle and high school students. Other than these two drugs, the lifetime prevalence estimates of the other illegal drugs or inhalants were relatively low.

In contrast to a gradual decline observed in the lifetime prevalence estimates, the incidence rates of alcohol (for both middle and high school students) and tobacco (for high school students) increased over the years (Additional file 1: Table 2). For illegal drugs or inhalants, incidences remained Additional file 1: Table during the 3-year period for middle school students, whereas a decreasing trend in the incidence of use of ecstasy, ketamine, marijuana, and GHB was found for high school students. Regardless of age (as indexed by middle and high schools), the pattern of ecstasy and ketamine incidence rates over three years generally mirrors that in the prevalence estimates.

**Figure 1:**
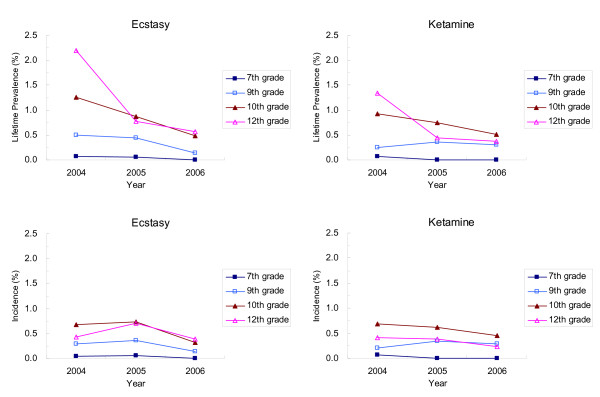
**Lifetime prevalence and incidence rate of ecstasy and ketamine use among school-attending adolescents, the 2006–06 National Survey of Illegal Drug Use among Adolescents (NSIDA) (*note: all percentages are weighted*)**.

The lifetime prevalence and incidence of ecstasy and ketamine are displayed in Figure 1. In general, the higher the grade, the higher the prevalence or incidence rate of drug use for both ecstasy and ketamine across years. A sharper decline in lifetime prevalence than in incidence rate for these two drugs among higher grade students (especially 12^th ^graders) was found from 2004 to 2005. For middle school students, both prevalence and incidence remained stably low.

On the basis of multivariable logistic regression analyses, Additional file 1: Table 3 displays those variables having a significant association with the incident use of ecstasy in any of three survey years. In general, the pattern of correlate estimates appeared different across survey years. For the first year, correlates of ecstasy use included those of moderate ORs (< 3), e.g., female gender and working experience, and those of high ORs (> 4), e.g., truancy, sexual experience, tobacco use, and betel nut use. For those with moderate ORs, the association disappeared (gender) or became diluted (working experience) as years went on. In contrast, weekly monetary allowance became significant only at the third year. Most of the correlates with high ORs at the first year remained consistently associated with incident use of ecstasy, including sexual experience, tobacco use, and betel nut use. One notable exception is truancy, which exhibited a significant decreasing association with ecstasy use over time.

The use of alcohol and tobacco (nearly 90%) was extremely common among ecstasy users, while that of betel nuts was around 40%. In terms of illegal drugs, use of ketamine (41.4–53.5%) was the most common, followed by marijuana (12.7–18.7%), and then methamphetamine (4.2–9.5%) (Additional file 1: Table 4). In comparing the ages at onset among drugs in the first survey year, more than half of ecstasy users had already used tobacco, alcohol, or betel nuts, and 10% or less of them started the use of these substances after the initiation of ecstasy use. However, the proportion of ecstasy users who initiated use of these substances either at the same age or after that of ecstasy continued to increase over the years, particularly for alcohol and tobacco. Half or more of the ecstasy users started using illegal drugs such as ketamine, marijuana, and methamphetamine at the same age as the ecstasy. However, the trend over the years was less stable for these illegal drugs probably due to the small number of users. Nevertheless, ketamine continued to be initiated almost exclusively at the same age as ecstasy, but the proportion of ecstasy-naïve ketamine users increased slightly but significantly from 0% in 2004 to 5.3% in 2006.

## Discussion

On the basis of nationally representative samples of school-attending adolescents in Taiwan, the estimates of illegal drug use, regardless of drug category, are far lower than those derived from industrialized countries. To illustrate, less than one in ten thousand adolescents had tried marijuana in Taiwan, in comparison with 20% and above in Australia, Europe, and the United States [41]. One of the major reasons accounting for this observation may be the severe punishments associated with illegal drug use in Taiwan. Before the lifting of martial law in 1987, sellers or users of narcotic drugs in Taiwan could face the death penalty. Even after a major amendment to the drug laws in 1998, first-time offenders of schedule I (e.g., heroin) or II (e.g.,, methamphetamine, ecstasy, and marijuana) controlled drugs are still considered as criminal patients and, therefore, are required to receive some form of detoxification or treatment in detention facilities [42]. Another possibility is that school-based surveys could not ascertain students with irregular school attendance, a subgroup at a higher risk of illegal drug involvement [35].

Ecstasy appeared to be the most commonly used illegal drug in middle- and high-schools from 2004 through 2006, with the exception of middle schools in 2006; but even so, a steady decline was noticed not only in its lifetime prevalence but also in incidence rate over the 3-year period. The secular changes observed in ecstasy prevalence and incidence in this study may provide a brief sketch of drug culture among Taiwanese adolescents. In 2004, the start of the three-year study, the prevalence of any illegal drug/inhalant use (1.42%) was similar to prevalences found in an earlier survey (1991–1996) among school-attending adolescents aged 13–18 years, ranging from 1.1% to 1.5% [30]. Despite this similarity, the nature of illegal drugs used by adolescents was quite different in these two periods. In the early 1990s, the most commonly consumed illegal drugs or inhalants in Taiwan were methamphetamine, sniffing glue, and flunitrazepam [30], whereas in this study the drug list changed to ecstasy, ketamine, and marijuana. In other words, rave-related drugs have become the drugs of choice for adolescent users in Taiwan since the early 2000s, a transition which may not be unique to Taiwan [43]. Our data over the 3-year period further indicated that there may be another evolution in the ecstasy epidemic reflected by a drop in both the lifetime prevalence and incidence rate. Intriguingly, a similar trend of decline in ecstasy use was also found in the recent national surveys in the U.S. [27], in which the lifetime prevalence of ecstasy use for 12^th ^graders decreased from 12% in 2001 to 6% in 2006.

The lowered prevalence and incidence of ecstasy in adolescents over the three years of study should be scrutinized within the context of changes in all psychoactive substances. On one hand, the lifetime prevalence estimates of many other substances were also in decrease, particularly for high school students, and included readily available substances (tobacco and betel nut) and illegal drugs related to raves or clubs (ketamine, marijuana, flunitrazepam, and GHB). On the other hand, some of those substances that had a decreasing trend in prevalence either exhibited an increasing trend (e.g., alcohol and tobacco), remained relatively stable (e.g., ecstasy for middle school students) or, to a lesser extent, decreased in onset rate (ecstasy, ketamine, and marijuana for high school students). The trends in both prevalence and incidence indicate that ecstasy has been steadily loosing its appeal among Taiwanese adolescents during the study period.

In contrast with a sharp decrease in ecstasy use, ketamine seems to be becoming more popular within adolescent drug culture, particularly among middle school students, despite that the two drugs were initiated at the same age for more than 85% of adolescent drug users. This might be attributed to harsher regulations influencing the use of ecstasy (offenders being incarcerated for enforced detoxification and drug education) compared with that of ketamine (no incarceration for offenders) in Taiwan. A number of studies have reported possible epidemic of ketamine misuse in many parts of the world [44,45], including Hong-Kong [11]. It is worthwhile to closely monitor the use of ketamine among adolescents. Finally, the trends for traditional illegal drugs or inhalants, such as methamphetamine and glue, appeared to be stable in both lifetime prevalence and incidence. Methamphetamine or glue use may characterize a subgroup of adolescents that is less sensible to alteration in drug cultures or market.

An examination of the correlates of incident ecstasy use revealed that there were common correlates of incident ecstasy use across years, including sexual experience, tobacco use, and betel nut use. Meanwhile, other sociobehavioral characteristics for ecstasy users appeared to change with time, such as truancy, female gender, working experience, and weekly allowance. In particular, the decreasing trend of the association of truancy with incident ecstasy use over the years seems to indicate that as ecstasy became less fashionable or available, partly due to drug dealers' promotion of lower-schedule drugs such as ketamine, the influx of experimental ecstasy users may be lowered. This may also explain why truancy alone was no longer a significant correlate in 2006 unless the adolescent had a large amount of disposable money.

A comparison of the student's ages at the onset of ecstasy use with that of other drugs indicates that there was no apparent gateway illegal drug that preceded ecstasy use except readily available substances such as tobacco, alcohol, or betel nut. This pattern is quite different from those of industrialized countries, where marijuana consistently has been found to be the first illegal drug used by adolescents [46], and almost one hundred percent of ecstasy users had used marijuana earlier in their lifetimes [14,28]. This is likely related to societal or culture-associated differences in drug availability and opportunity for exposure [47].

Our results should be interpreted with some limitations in mind. First, the change in the mode of administration from paper-and-pencil to web-based questionnaire in the fall of the first year makes the first year's estimates for high- and vocational-school students not directly comparable to the counterparts of the second and third years. Our previous study indicated that the web-based questionnaire had a higher reporting rate than paper-and-pencil questionnaire [34], and thus, the magnitude of decrease in ecstasy use would be underestimated rather than overestimated. Second, the small number of ecstasy users, due to a low prevalence, limits our ability to perform precise analyses to probe drug careers in more details (e.g., survival analyses). Nevertheless, we did estimate incident use that was initiated in the past year capturing the onset use in each year's survey. Third, our definition of incident use of substance would underestimate the incidence rate given the cross-sectional nature of the survey, i.e., about half of the students who initiated use within the past year reported that they were one year younger at initiation (assuming birthdays are evenly distributed throughout the year). Finally, there might be some emerging trend of drug use not captured in our survey, such as non-medical use of prescription drugs, which was not assessed in this study.

## Conclusion

In conclusion, ecstasy by and large was the most commonly used illegal drug among Taiwanese middle and high school students from 2004 through 2006; however, a steady decline was noticed in its prevalence and onset rate over the study period. Having sexual experience, tobacco use, and betel nut use were consistently associated with onset of ecstasy use across years. Additionally, the associations linking ecstasy use with truancy, gender, and working experience weakened or diminished with time, whereas the association with weekly monetary allowance became more salient in the third year. The majority of ecstasy users had the experience of polydrug use, with ketamine being the most commonly experienced illegal drug. The initiation age of ecstasy use preceded that of the other illegal drugs. Although ecstasy has been the leading illegal drug used by school-attending adolescents in Taiwan since the early 2000s, its prevalence and incidence rate has been declining. A notable increasing trend of ketamine use warrants more attention in the future.

## Competing interests

The authors declare that they have no competing interests.

## Authors' contributions

WJC was the principal investigator, contributed to the conceptualization of the original research, oversaw all aspects of its implementation, and led the writing of the article. TCF and GMT supervised the fieldwork of the survey and assisted data management and analysis. TTT and WLH were responsible for data analyses and preparation of the tables. CKH assisted with the sampling design and data management. CYC assisted data analyses, help interpret the results and edit the manuscript. All authors contributed to and have approved the final manuscript.

## Pre-publication history

The pre-publication history for this paper can be accessed here:



## Supplementary Material

Additional file 1 **Tables 1–4.** These tables are in landscape pages and are referred to in main text. Click here for file
