# Supplementary material for: Use of ecstasy and other psychoactive substances among school-attending adolescents in Taiwan: national surveys 2004–2006
Source: BMC Public Health. 2009 Jan 21;9:27. doi: 10.1186/1471-2458-9-27 (PMC2636802; doi:10.1186/1471-2458-9-27)
Supplement: Additional file 1 — Tables 1–4. These tables are in landscape pages and are referred to in main text. [file 1471-2458-9-27-S1.doc]

**Table 1.** Lifetime prevalence of psychoactive substance use among school-attending adolescents, the 2004-2006 National Survey of Illegal Drug Use among Adolescents (NSIDA).

|  | 2004 | | |  | 2005 | | |  | 2006 | | |  | Cochran-Armitage | |
| --- | --- | --- | --- | --- | --- | --- | --- | --- | --- | --- | --- | --- | --- | --- |
|  | (N = 7083) | | |  | (N = 6836) | | |  | (N = 6172) | | |  | trend test in prevalence | |
| Lifetime use of  Substance | n | %_wt_ (SE)^a^ | Age at  first use (SD) |  | n | %_wt_ (SE)^a^ | Age at  first use (SD) |  | n | %_wt_ (SE)^a^ | Age at  first use (SD) |  | Statistic^b^ | ′06 −′04  change (%) |
| *Middle school students (grades 7 and 9)* | | |  |  |  |  |  |  |  |  |  |  |  |  |
| Readily available substance |  |  |  |  |  |  |  |  |  |  |  |  |  |  |
| Alcohol | 1779 | 24.35 (1.19) | 11.9 (2.7) |  | 1726 | 24.98 (1.33) | 13.1 (2.6) |  | 1478 | 23.22 (1.40) | 13.0 (2.7) |  | 1.45 | - |
| Tobacco | 889 | 12.04 (1.03) | 11.6 (2.7) |  | 728 | 10.28 (0.97) | 13.0 (2.6) |  | 618 | 9.83 (0.85) | 12.9 (2.7) |  | 4.17*** | −18.4 |
| Betel nut | 221 | 2.68 (0.27) | 11.4 (2.8) |  | 181 | 2.49 (0.30) | 12.8 (3.0) |  | 119 | 1.77 (0.26) | 12.9 (2.5) |  | 3.41** | −34.0 |
| Illegal drug or inhalant |  |  |  |  |  |  |  |  |  |  |  |  |  |  |
| Ecstasy | 23 | 0.28 (0.10) | 13.5 (1.3) |  | 20 | 0.25 (0.06) | 14.3 (2.7) |  | 3 | 0.07 (0.04) | 14.3 (1.2) |  | 2.64* | −75.0 |
| Ketamine | 14 | 0.15 (0.05) | 14.4 (0.8) |  | 15 | 0.18 (0.05) | 13.9 (2.9) |  | 10 | 0.15 (0.06) | 15.4 (0.8) |  | -0.02 | - |
| Marijuana | 4 | 0.07 (0.04) | 12.8 (2.8) |  | 3 | 0.03 (0.02) | 12.3 (6.4) |  | 1 | 0.02 (0.02) | 13.0 ( - ) |  | 1.35 | - |
| Glue | 14 | 0.19 (0.05) | 11.9 (1.5) |  | 16 | 0.26 (0.07) | 12.0 (3.4) |  | 14 | 0.19 (0.07) | 10.9 (2.9) |  | -0.05 | - |
| Methamphetamine | 5 | 0.05 (0.03) | 11.7 (1.5) |  | 8 | 0.09 (0.03) | 12.3 (3.9) |  | 3 | 0.05 (0.03) | 14.7 (2.3) |  | 0.00 | - |
| Flunitrazepam | 4 | 0.05 (0.03) | 14.0 (2.0) |  | 6 | 0.07 (0.03) | 13.2 (3.9) |  | 2 | 0.03 (0.02) | 16.0 ( - ) |  | 0.43 | - |
| GHB | 5 | 0.07 (0.04) | 14.0 (0.0) |  | 2 | 0.02 (0.01) | 14.0 (1.4) |  | 0 | 0 | - |  | 2.38* | −100.0 |
| Heroin | 9 | 0.15 (0.05) | 12.2 (2.3) |  | 9 | 0.12 (0.04) | 13.6 (1.5) |  | 7 | 0.12 (0.04) | 12.9 (2.2) |  | 0.59 | - |
| Angel dust | 0 | 0 | - |  | 2 | 0.03 (0.02) | 15.5 (0.7) |  | 0 | 0 | - |  | -0.08 | - |
| Any illegal drug/inhalant | 50 | 0.65 (0.12) | 11.6 (3.6) |  | 52 | 0.73 (0.11) | 12.8 (3.1) |  | 35 | 0.53 (0.13) | 12.9 (2.9) |  | 0.80 | - |

^a^Estimated percentages were derived based on complex survey analyses (weighted data with Taylor series linearization).

^b^Two-sided test for the weighted prevalence estimates.

*p < 0.05, **p < 0.01, ***p < 0.001 (To be continued)

**Table 1.** (Continued) Lifetime prevalence of psychoactive substance use among school-attending adolescents, the 2004-2006 National Survey of Illegal Drug Use among Adolescents (NSIDA).

|  | 2004 | | |  | 2005 | | |  | 2006 | | |  | Cochran-Armitage | |
| --- | --- | --- | --- | --- | --- | --- | --- | --- | --- | --- | --- | --- | --- | --- |
|  | (N = 11149) | | |  | (N = 11150) | | |  | (N = 11692) | | |  | trend test in prevalence | |
| Lifetime use of  Substance | n | %wt (SE)^a^ | Age at  first use (SD) |  | n | %wt (SE)^a^ | Age at  first use (SD) |  | n | %wt (SE)^a^ | Age at  first use (SD) |  | Statistic^b^ | ′06 −′04  change (%) |
| *High school students (grades 10 and 12)* | | |  |  |  |  |  |  |  |  |  |  |  |  |
| Readily available substance |  |  |  |  |  |  |  |  |  |  |  |  |  |  |
| Alcohol | 4521 | 43.08 (1.88) | 13.9 (2.8) |  | 4792 | 42.14 (0.87) | 15.1 (3.0) |  | 5022 | 42.59 (0.95) | 15.3 (2.8) |  | 0.74 | - |
| Tobacco | 2777 | 25.84 (1.58) | 13.2 (2.6) |  | 2217 | 19.24 (0.75) | 14.5 (2.9) |  | 2141 | 17.76 (0.91) | 14.5 (2.8) |  | 14.95*** | −32.5 |
| Betel nut | 767 | 7.32 (0.92) | 13.6 (3.0) |  | 501 | 4.28 (0.35) | 14.7 (3.2) |  | 384 | 3.26 (0.50) | 14.5 (3.4) |  | 14.13*** | −55.5 |
| Illegal drug or inhalant |  |  |  |  |  |  |  |  |  |  |  |  |  |  |
| Ecstasy | 149 | 1.72 (0.53) | 15.5 (2.0) |  | 96 | 0.82 (0.14) | 16.6 (1.8) |  | 68 | 0.52 (0.08) | 16.0 (2.6) |  | 9.06*** | −70.0 |
| Ketamine | 105 | 1.13 (0.29) | 15.9 (1.5) |  | 72 | 0.61 (0.12) | 16.8 (1.4) |  | 55 | 0.44 (0.08) | 16.0 (2.0) |  | 6.10*** | −61.1 |
| Marijuana | 39 | 0.48 (0.15) | 15.6 (1.3) |  | 31 | 0.27 (0.05) | 17.1 (1.7) |  | 14 | 0.09 (0.03) | 15.5 (2.4) |  | 5.60*** | −81.3 |
| Glue | 23 | 0.16 (0.04) | 12.6 (3.1) |  | 10 | 0.10 (0.03) | 13.3 (3.2) |  | 11 | 0.08 (0.03) | 12.8 (3.0) |  | 1.71 | - |
| Methamphetamine | 15 | 0.12 (0.04) | 15.1 (1.7) |  | 11 | 0.07 (0.03) | 16.0 (1.1) |  | 9 | 0.06 (0.03) | 15.1 (1.8) |  | 1.53 | - |
| Flunitrazepam | 13 | 0.13 (0.04) | 15.0 (1.2) |  | 6 | 0.07 (0.03) | 15.7 (2.9) |  | 2 | 0.02 (0.02) | 15.5 (0.7) |  | 3.04** | −84.6 |
| GHB | 9 | 0.15 (0.10) | 15.6 (1.2) |  | 4 | 0.03 (0.02) | 14.3 (5.2) |  | 3 | 0.01 (0.01) | 15.7 (0.6) |  | 4.06*** | −93.3 |
| Heroin | 4 | 0.03 (0.02) | 13.5 (2.5) |  | 3 | 0.02 (0.01) | 14.0 (2.6) |  | 3 | 0.02 (0.02) | 10.7 (6.5) |  | 0.36 | - |
| Angel dust | 2 | 0.02 (0.01) | 14.5 (0.7) |  | 2 | 0.02 (0.02) | 14.5 (3.5) |  | 0 | 0 | - |  | 1.21 | - |
| Any illegal drug/inhalant | 225 | 2.31 (0.50) | 15.2 (2.2) |  | 154 | 1.33 (0.17) | 16.3 (2.2) |  | 103 | 0.80 (0.11) | 15.5 (2.8) |  | 9.4*** | −65.4 |

^a^Estimated percentages were derived based on complex survey analyses (weighted data with Taylor series linearization).

^b^Two-sided test for the weighted prevalence estimates.

*p < 0.05, **p < 0.01, ***p < 0.001

**Table 2.** Estimated incidence rate of psychoactive substance initiation, the 2004-2006 National Survey of Illegal Drug Use among Adolescents (NSIDA).

|  | 2004 | | |  | 2005 | | |  | 2006 | | |  | Cochran-Armitage | |
| --- | --- | --- | --- | --- | --- | --- | --- | --- | --- | --- | --- | --- | --- | --- |
|  | (N = 7083) | | |  | (N = 6836) | | |  | (N = 6172) | | |  | trend test in incidence | |
| Incident use of  Substance | n | %_wt_ (SE)^a^ | Age at  first use (SD) |  | n | %_wt_ (SE)^a^ | Age at  first use (SD) |  | n | %_wt_ (SE)^a^ | Age at  first use (SD) |  | Statistic^b^ | ′06 −′04  change (%) |
| *Middle school students (grades 7 and 9)* | | |  |  |  |  |  |  |  |  |  |  |  |  |
| Readily available substance |  |  |  |  |  |  |  |  |  |  |  |  |  |  |
| Alcohol | 1029 | 13.97 (0.73) | 13.5 (1.2) |  | 1255 | 18.08 (1.03) | 14.2 (1.4) |  | 1050 | 16.35 (0.99) | 14.1 (1.3) |  | -3.94*** | +17.0 |
| Tobacco | 465 | 6.49 (0.60) | 13.4 (1.2) |  | 489 | 6.97 (0.70) | 14.2 (1.3) |  | 405 | 6.47 (0.62) | 14.1 (1.3) |  | -0.01 | - |
| Betel nut | 105 | 1.36 (0.20) | 13.4 (1.2) |  | 111 | 1.61 (0.21) | 14.5 (1.3) |  | 77 | 1.21 (0.21) | 14.0 (1.4) |  | 0.64 | - |
| Illegal drug or inhalant |  |  |  |  |  |  |  |  |  |  |  |  |  |  |
| Ecstasy | 15 | 0.17 (0.05) | 14.1 (0.8) |  | 17 | 0.21 (0.05) | 14.7 (1.2) |  | 3 | 0.07 (0.04) | 14.3 (1.2) |  | 1.38 | - |
| Ketamine | 11 | 0.13 (0.05) | 14.3 (0.8) |  | 14 | 0.17 (0.05) | 14.6 (0.9) |  | 9 | 0.15 (0.06) | 15.2 (0.7) |  | -0.25 | - |
| Marijuana | 2 | 0.02 (0.01) | 12.5 (2.1) |  | 1 | 0.01 (0.01) | 16.0 ( - ) |  | 1 | 0.02 (0.02) | 13.0 ( - ) |  | 0.03 | - |
| Glue | 3 | 0.04 (0.03) | 12.7 (1.5) |  | 9 | 0.15 (0.06) | 13.6 (1.4) |  | 6 | 0.09 (0.04) | 13.2 (1.8) |  | -0.89 | - |
| Methamphetamine | 0 | 0.00 (0.00) | - |  | 3 | 0.02 (0.01) | 14.3 (1.2) |  | 2 | 0.04 (0.03) | 16.0 (0.0) |  | -1.76 | - |
| Flunitrazepam | 3 | 0.04 (0.02) | 13.7 (2.3) |  | 2 | 0.04 (0.03) | 14.5 (2.1) |  | 1 | 0.01 (0.01) | 16.0 ( - ) |  | 0.78 | - |
| GHB | 2 | 0.04 (0.03) | 14.0 (0.0) |  | 2 | 0.02 (0.01) | 14.0 (1.4) |  | 0 | 0 | - |  | 1.62 | - |
| Heroin | 3 | 0.06 (0.03) | 13.7 (1.5) |  | 6 | 0.07 (0.03) | 14.0 (1.7) |  | 5 | 0.07 (0.03) | 13.8 (1.8) |  | -0.37 | - |
| Angel dust | 0 | 0 | - |  | 0 | 0 | - |  | 0 | 0 | - |  | - | - |
| Any illegal drug/inhalant | 26 | 0.32 (0.07) | 13.7 (1.4) |  | 36 | 0.50 (0.09) | 14.1 (1.3) |  | 22 | 0.35 (0.10) | 14.3 (1.6) |  | -0.34 | - |

^a^Estimated percentages were derived based on complex survey analyses (weighted data with Taylor series linearization).

^b^Two-sided test for the weighted incidence estimates.

*p < 0.05, **p < 0.01, ***p < 0.001 (To be continued)

**Table 2.** (Continued) Estimated incidence rate of psychoactive substance initiation, the 2004-2006 National Survey of Illegal Drug Use among Adolescents (NSIDA).

|  | 2004 | | |  | 2005 | | |  | 2006 | | |  | Cochran-Armitage | |
| --- | --- | --- | --- | --- | --- | --- | --- | --- | --- | --- | --- | --- | --- | --- |
|  | (N = 11149) | | |  | (N = 11150) | | |  | (N = 11692) | | |  | trend test in incidence | |
| Incident use of  Substance | n | %_wt_ (SE)^a^ | Age at  first use (SD) |  | n | %_wt_ (SE)^a^ | Age at  first use (SD) |  | n | %_wt_ (SE)^a^ | Age at  first use (SD) |  | Statistic^b^ | ′06 −′04  change (%) |
| *High school students (grades 10 and 12)* | | |  |  |  |  |  |  |  |  |  |  |  |  |
| Readily available substance |  |  |  |  |  |  |  |  |  |  |  |  |  |  |
| Alcohol | 1471 | 13.51 (0.45) | 16.2 (1.1) |  | 2915 | 25.54 (0.70) | 16.8 (1.2) |  | 3172 | 26.08 (0.71) | 16.8 (1.3) |  | -22.84*** | +93.1 |
| Tobacco | 540 | 4.35 (0.56) | 15.9 (1.0) |  | 1046 | 9.04 (0.48) | 16.4 (1.2) |  | 1030 | 8.41 (0.54) | 16.4 (1.2) |  | -11.68*** | +93.3 |
| Betel nut | 199 | 1.76 (0.25) | 16.0 (1.0) |  | 270 | 2.43 (0.25) | 16.8 (1.2) |  | 208 | 1.81 (0.34) | 16.6 (1.2) |  | -0.23 | - |
| Illegal drug or inhalant |  |  |  |  |  |  |  |  |  |  |  |  |  |  |
| Ecstasy | 78 | 0.55 (0.10) | 16.3 (1.1) |  | 83 | 0.72 (0.13) | 16.9 (1.3) |  | 49 | 0.35 (0.07) | 16.7 (1.5) |  | 2.08* | −36.4 |
| Ketamine | 71 | 0.55 (0.12) | 16.4 (1.1) |  | 64 | 0.51 (0.09) | 17.0 (1.3) |  | 41 | 0.35 (0.08) | 16.3 (1.4) |  | 2.28* | −36.4 |
| Marijuana | 17 | 0.15 (0.05) | 16.3 (1.0) |  | 26 | 0.24 (0.05) | 17.3 (1.1) |  | 7 | 0.05 (0.02) | 16.4 (1.0) |  | 1.97* | −66.7 |
| Glue | 3 | 0.02 (0.01) | 16.3 (1.2) |  | 3 | 0.04 (0.02) | 15.7 (1.2) |  | 2 | 0.01 (0.00) | 17.0 (1.4) |  | 0.68 | - |
| Methamphetamine | 5 | 0.03 (0.02) | 16.2 (0.8) |  | 7 | 0.05 (0.02) | 16.3 (1.3) |  | 8 | 0.06 (0.03) | 15.6 (1.1) |  | -0.99 | - |
| Flunitrazepam | 4 | 0.04 (0.02) | 16.3 (1.0) |  | 5 | 0.06 (0.03) | 16.8 (0.8) |  | 1 | 0.01 (0.01) | 15.0 ( - ) |  | 1.03 | - |
| GHB | 6 | 0.12 (0.10) | 16.0 (0.9) |  | 2 | 0.02 (0.01) | 18.5 (0.7) |  | 3 | 0.01 (0.01) | 15.7 (0.6) |  | 3.61*** | −91.7 |
| Heroin | 1 | 0.01 (0.01) | 16.0 ( - ) |  | 1 | 0.01 (0.01) | 17.0 ( - ) |  | 1 | 0 | 17.0 ( - ) |  | 0.38 | - |
| Angel dust | 0 | 0 | - |  | 1 | 0.01 (0.01) | 17.0 ( - ) |  | 0 | 0 | - |  | 0.02 | - |
| Any illegal drug/inhalant | 127 | 1.03 (0.15) | 16.0 (1.3) |  | 129 | 1.12 (0.14) | 16.7 (1.4) |  | 72 | 0.54 (0.09) | 16.5 (1.4) |  | 4.04*** | −47.6 |

^a^Estimated percentages were derived based on complex survey analyses (weighted data with Taylor series linearization).

^b^Two-sided test for the weighted incidence estimates.

*p < 0.05, **p < 0.01, ***p < 0.001

**Table 3.** Estimated association linking potential correlates with incident use of ecstasy among ecstasy-naïve school-attending adolescents, the 2004-2006 NSIDA.

|  | 2004 (N = 18152) | | |  | 2005 (N = 17970) | | |  | 2006 (N = 17841) | | |  | Trend in ORs with year^d^ |
| --- | --- | --- | --- | --- | --- | --- | --- | --- | --- | --- | --- | --- | --- |
| Variable^a^ | N | n (%_wt_) ^b^ | aOR^c^ (95% CI) |  | N | n (%_wt_) ^b^ | aOR^c^ (95% CI) |  | N | n (%_wt_) ^b^ | aOR^c^ (95% CI) |  | Z-score |
| Gender |  |  |  |  |  |  |  |  |  |  |  |  |  |
| Female | 8693 | 43 (0.35) | 1.00 |  | 8878 | 47 (0.40) | 1.00 |  | 9102 | 31 (0.19) | 1.00 |  |  |
| Male | 9416 | 50 (0.32) | 0.34 (0.16-0.71)** |  | 9091 | 53 (0.45) | 0.72 (0.36-1.44) |  | 8739 | 21 (0.19) | 0.46 (0.15-1.38) |  | 0.34 |
| Working experience |  |  |  |  |  |  |  |  |  |  |  |  |  |
| No | 16531 | 68 (0.24) | 1.00 |  | 16596 | 68 (0.29) | 1.00 |  | 16742 | 38 (0.17) | 1.00 |  |  |
| Yes | 1281 | 23 (1.76) | 2.34 (1.23-4.44)** |  | 1242 | 29 (2.65) | 1.83 (1.10-3.07)* |  | 1089 | 14 (0.82) | 1.13 (0.48-2.65) |  | 1.91 |
| Weekly allowance |  |  |  |  |  |  |  |  |  |  |  |  |  |
| 0 – 500 | 12136 | 34 (0.18) | 1.00 |  | 12188 | 43 (0.31) | 1.00 |  | 12219 | 20 (0.09) | 1.00 |  |  |
| 501 or above | 5374 | 55 (0.70) | 1.43 (0.71-2.87) |  | 5752 | 57 (0.75) | 1.03 (0.62-1.70) |  | 5575 | 32 (0.52) | 2.98 (1.32-6.76)** |  | 1.64 |
| Truancy |  |  |  |  |  |  |  |  |  |  |  |  |  |
| No | 13426 | 10 (0.03) | 1.00 |  | 13738 | 15 (0.09) | 1.00 |  | 13753 | 9 (0.04) | 1.00 |  |  |
| Yes | 4610 | 83 (1.45) | 15.99 (5.43-47.02)*** |  | 4225 | 85 (1.83) | 5.41 (2.30-12.68)*** |  | 4043 | 43 (0.85) | 2.76 (0.94-8.06) |  | 5.18* |
| Sexual experience |  |  |  |  |  |  |  |  |  |  |  |  |  |
| No | 16230 | 30 (0.11) | 1.00 |  | 16937 | 40 (0.18) | 1.00 |  | 16792 | 17 (0.07) | 1.00 |  |  |
| Yes | 1796 | 62 (2.34) | 7.30 (3.49-15.29)*** |  | 1026 | 59 (6.05) | 6.43 (3.64-11.35)*** |  | 1046 | 35 (2.89) | 8.14 (3.39-19.55)*** |  | 0.05 |
| Alcohol use |  |  |  |  |  |  |  |  |  |  |  |  |  |
| No | 11885 | 10 (0.11) | 1.00 |  | 11466 | 12 (0.06) | 1.00 |  | 11358 | 8 (0.04) | 1.00 |  |  |
| Yes | 6224 | 83 (0.82) | 0.60 (0.19-1.92) |  | 6503 | 88 (1.20) | 3.89 (1.54-9.86)** |  | 6481 | 44 (0.52) | 1.64 (0.40-6.74) |  | 1.52 |
| Tobacco use |  |  |  |  |  |  |  |  |  |  |  |  |  |
| No | 14517 | 7 (0.03) | 1.00 |  | 15038 | 12 (0.07) | 1.00 |  | 15100 | 5 (0.03) | 1.00 |  |  |
| Yes | 3592 | 86 (1.78) | 13.12 (3.64-47.31)*** |  | 2931 | 88 (2.59) | 4.63 (1.40-15.35)* |  | 2741 | 47 (1.26) | 6.70 (1.27-35.47)* |  | 0.58 |
| Betel nut use |  |  |  |  |  |  |  |  |  |  |  |  |  |
| No | 17160 | 53 (0.20) | 1.00 |  | 17292 | 57 (0.27) | 1.00 |  | 17343 | 30 (0.11) | 1.00 |  |  |
| Yes | 949 | 40 (3.26) | 4.80 (2.55-9.06)*** |  | 677 | 43 (5.04) | 2.96 (1.57-5.59)*** |  | 498 | 22 (3.44) | 5.92 (2.64-13.25)*** |  | 0.04 |

^a^ Only those variables that were associated with incident use of ecstasy in multivariable logistic regression analyses for at least year were displayed here.

^b^Estimated percentages were derived based on complex survey analyses (weighted data with Taylor series linearization).

^c^Adjusted odds ratio, estimated from a model containing region, age, family structure, and all the variables in this table.

^d^By means of combining all the three years’ data and adding an interaction term between each covariate and survey year (coded as 0, 1, and 2); Wald test of the coefficient of the interaction term was used to assess the trend in odds ratio over years.

* P<0.05, ** P<0.01, *** P<0.001 for the test.

**Table 4.** Temporal sequence in onset ages of ecstasy in relation to other psychoactive substances among school-attending adolescents with ecstasy experiences, the 2004-06 NSIDA.

|  | 2004  (N = 166) | | | |  | 2005  (N = 116) | | | |  | 2006  (N = 71) | | | |  | Jonckheere-Terpstra trend test | |
| --- | --- | --- | --- | --- | --- | --- | --- | --- | --- | --- | --- | --- | --- | --- | --- | --- | --- |
| Substance | n  (n/N %) | Younger (%) | Same age (%) | Older (%) |  | n  (n/N %) | Younger (%) | Same age (%) | Older (%) |  | n  (n/N %) | Younger (%) | Same age (%) | Older (%) |  | Z | p-value^a^ |
| Alcohol | 152 (91.6) | 71.1 | 23.0 | 5.9 |  | 103 (88.8) | 67.0 | 29.1 | 3.9 |  | 62 (87.3) | 54.8 | 29.0 | 16.1 |  | 2.2 | 0.02* |
| Tobacco | 153 (92.2) | 72.6 | 22.2 | 5.2 |  | 102 (87.9) | 75.5 | 19.6 | 4.9 |  | 64 (90.1) | 60.9 | 28.1 | 10.9 |  | 1.3 | 0.20 |
| Betel nut | 74 (44.6) | 60.8 | 31.1 | 8.1 |  | 48 (41.4) | 54.2 | 37.5 | 8.3 |  | 27 (38.0) | 29.6 | 51.9 | 18.5 |  | 2.5 | 0.01* |
| Ketamine | 71 (42.8) | 0.0 | 85.9 | 14.1 |  | 48 (41.4) | 4.2 | 93.8 | 2.1 |  | 38 (53.5) | 5.3 | 86.8 | 7.9 |  | -2.2 | 0.03* |
| Marijuana | 31 (18.7) | 12.9 | 51.6 | 35.5 |  | 15 (12.9) | 13.3 | 73.3 | 13.3 |  | 9 (12.7) | 11.1 | 77.8 | 11.1 |  | -1.4 | 0.16 |
| Methamphetamine | 7 (4.2) | 14.3 | 71.4 | 14.3 |  | 11 (9.5) | 36.4 | 45.5 | 18.2 |  | 6 (8.50 | 0.0 | 83.3 | 16.7 |  | 0.4 | 0.69 |

^a^Two-sided, assessing the trend of the trichotomous proportions (younger, same age, and older) by survey year.

*p<0.05
